# Facile One-Pot Synthesis and Anti-Microbial Activity of Novel 1,4-Dihydropyridine Derivatives in Aqueous Micellar Solution under Microwave Irradiation

**DOI:** 10.3390/molecules29051115

**Published:** 2024-03-01

**Authors:** Asmita Goswami, Navneet Kaur, Manvinder Kaur, Kishanpal Singh, Harvinder Singh Sohal, Haesook Han, Pradip K. Bhowmik

**Affiliations:** 1 Medicinal and Natural Product Laboratory, Department of Chemistry, Chandigarh University, Mohali 140413, Punjab, India; asmitagoswami3@gmail.com (A.G.);; 2 Department of Chemistry, Punjabi University, Patiala 147002, Punjab, India; kishanpal.3112@gmail.com; 3 Department of Chemistry and Biochemistry, University of Nevada Las Vegas, 4505 S. Maryland Parkway, Box 454003, Las Vegas, NV 89154, USA; hanh3@unlv.nevada.edu (H.H.);

**Keywords:** multi-component reaction, 1,4-dihydropyridines, microwave, heterocyclic compounds, Al(DS)_3_, green synthesis

## Abstract

The current study describes a novel and eco-conscious method to synthesize 1,4-dihydropyridine derivatives utilizing an aqueous micellar solution containing aluminum dodecyl sulfate, Al(DS)_3_, using readily available starting material. The final products were synthesized with excellent yields within remarkably quick reaction durations, promoting remarkable atom economy and minimizing environmental impacts. The present protocol has several advantages over other methodologies in terms of high yield (up to 97%) with excellent purity. Further, the synthesized 1,4-DHPs exhibit favorable to excellent resistance against examined bacterial and fungal species. Intriguingly, polar groups on the phenyl ring (**5b**, **5c**, **5i** and **5j**) make the 1,4-DHPs equally potent against the microbes as compared to the standard drugs.

## 1. Introduction

Researchers have long been intrigued by the biological properties of 1,4-dihydropyridines (1,4-DHPs). 1,4-DHPs constitute a significant category within the N-heterocyclic ring, consisting of a biologically vital nucleus that exhibits various important pharmacological properties and is used as an antibacterial, anticancer [1], anticoagulant, antileishmanial, anticonvulsant, antitubercular [2], antioxidant [3], antiulcer, antifertility, neuroprotection properties, antimalarials, HIV-1 protease inhibitor, antihypertensive [4], anti-atherosclerotic, hepato-protective, vasodilator, anti-mutagenic, bronchodilator, geroprotective, anti-tumor [5], and anti-diabetic agent [6]. Moreover, they constitute an essential category of agents that modulate calcium channels and have been widely employed in the treatment of cardiovascular disorders, exhibiting functions such as antihypertensive, antianginal, vasodilator, and cardiac depressant activities [7]. 1,4-DHP attracts more attention, due to its bioactivity and presence in coenzymes, diphosphate pyridine nucleotide (DPNH). In the pharmaceutical field, DHPs are already commercialized as amlodipine, felodipine, isradipine [8], lacidipine [9], nicardipine [10], nitrendipine [11], nifedipine, and nemadipine B [12], among them, nitrendipine and nemadipine B stand out for their potent calcium channel blocking activities.

In general, 1,4-DHPs are synthesized using multicomponent reactions, where a combination of β-keto-ester, aldehyde, as well as ammonia undergoes reaction to produce the required DHPs. Arthur Hantzsch described the first one-pot formation of symmetrically substituted 1,4-DHP in the year 1882 [13]. The Hantzsch reaction proved to be the most widely used protocol to access 1,4-DHPs and its numerous biologically active derivatives [14]. But due to drawbacks *such as* harsh reaction conditions, long reaction time, and generally low yield of products, new modifications were made to it to obtain the targeted compounds,1,4-DHPs, in good to excellent yields [15]. It has been reported that in multicomponent reactions (MCRs), a wide variety of catalysts has been studied for the production of 1,4-DHP derivatives, encompassing various Lewis or Bronsted acids *such as* Y(OTf)_3_ [16], free nano-Fe_2_O_3_ [17], TMU-33 [18], InCl_3_ [19]; heterogeneous catalyst *such as* HClO_4_–SiO_2_ [20], PdRuNi@GO NPs [7], zeolite [21], IRMOF-3 [22]; organ catalyst, CAN [23], Montmorillonite K10 clay [24], HClO_4_-SiO_2_ [25], tetrabutylammonium hydrogen sulfate, sodium- and Cs-Norit carbons, fermenting Baker’s yeast [26], metal triflates with the incorporation of numerous methodologies *such as* stirring [2], conventional heating [27], refluxing [22], microwave [1], visible light [4], and ultrasound irradiations [21]. Researchers are now focusing on the green aspects of this reaction leading to the usage of various green solvents such as glycerol [18], water [19], and ionic liquids [6]. Unless otherwise stated, aqueous surfactant solutions, as well as neat conditions, have also been incorporated to obtain the required products.

Persisting on our work, in the present work, we describe the synthesis of novel 1,4-DHP scaffolds using a one-pot method using an aqueous micellar solution as a greener solvent. The microwave-assisted reaction method stands out as superior due to its rapid reaction times, cost-effectiveness, and straightforward operational procedures compared to other methods. To begin our investigation into a sustainable and effective approach for synthesizing 1,4-DHPs, we commenced by employing a catalytic quantity of Al(DS)_3_ with a combination of substituted benzaldehyde, diethyl acetylene dicarboxylate, and either ammonium acetate or aniline, along with malononitrile under microwave irradiations at ambient temperature.

## 2. Result and Discussion

### 2.1. Characterization of Aluminum Dodecyl Sulfate, Al(DS)_3_

#### 2.1.1. SEM Characterization

**Figure 1:**
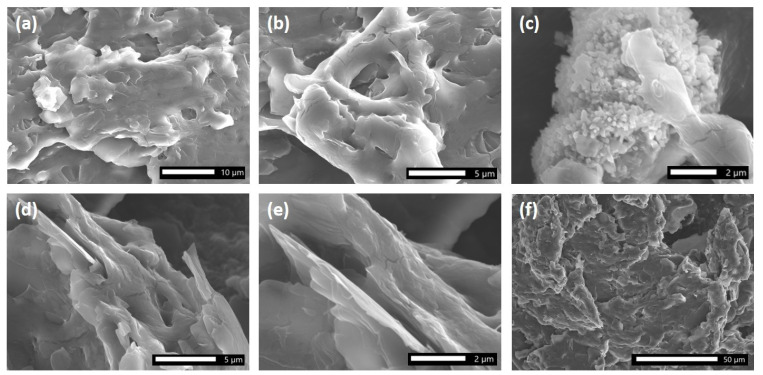
SEM images of Al(DS)_3_.

The SEM images depicted in Figure 1a–f, magnified from 500 to 7500 times, provide valuable insights into the morphology of the Al(DS)_3_ surfactant. This compound was produced as dense aggregates (Figure 1a) which are irregular in shape. At higher magnifications, it becomes evident that randomly organized microplates arranged to compose the micro-objects are in an edge-to-face style (Figure 1a–d).

Based on the elemental investigation conducted on microscopic sectors of Al(DS)_3_ using EDS (as shown in Figure S1), the atomic percentages of Al and S were determined to be 22.40% and 77.60%, respectively, which indicates a 1:3 stoichiometry.

#### 2.1.2. XRD Characterization

**Figure 2:**
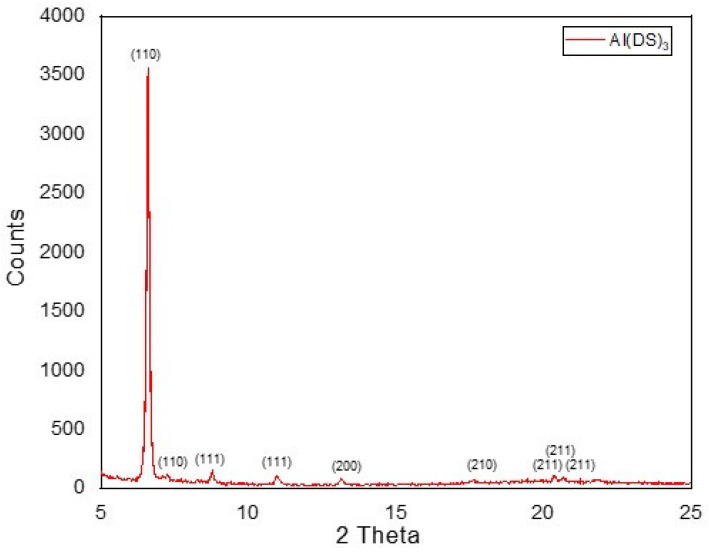
XRD images of Al(DS)_3_.

The XRD patterns in the range of 2θ = 5–25° for the Al(DS)_3_ sample are shown in Figure 2. The prepared sample of Al(DS)3 gave rise to XRD peaks at 2θ = 6.596, 7.184, 10.965, 13.166, 17.587, 20.387, 20.674, and 21.875, corresponding to (110), (110), (111), (111), (200), (210), (211), (211), and (211) diffraction peaks, respectively. According to the above data, the XRD patterns of Al(DS)_3_ show a prominent diffraction peak at (110) with preferential orientation around 2θ = 6.596° with 100% relative intensity, which shows a reasonable degree of crystallinity of 65.3%.

### 2.2. Preparation of [1,4-DHPs] 10-Amino-3,3,6,6-Tetramethyl-9-Aryl-3,4,6,7,9,10-Hexa-Hydroacridine-1,8(2H,5H)-Dione Derivatives (**5a**–**n**) Using Al(DS)_3_

Various solvents, including water, ethanol, ethanolic solution of *p*-TSA, glycerol, aqueous solution of SDS, and aqueous solution Al(DS)_3_, were employed for the condensation of benzaldehyde **1**, dialkyl acetylene dicarboxylate **2**, ammonium acetate **3**, and malononitrile **4**. Among these, water as a solvent proved to be the most effective, as indicated in Table 1. The maximum yield was achieved through an Al(DS)_3_ + water catalyst system.

Table 2 illustrates the optimal temperature for a reaction carried out in an aqueous micellar solution and it was also observed that increasing the temperature has a significant increase in yield with time. Conversely, further temperature increases, result in a decrease in product yield as the products start decomposing at higher temperatures.

Similarly, various other aldehydes **3b**–**g** will undergo reactions with diethyl acetylene dicarboxylate, malononitrile, and either ammonium acetate or aniline using the same procedure, with the formation monitored by TLC and melting point analysis. The reactions proceed smoothly even in the company of various electron-donating (-Me and -OMe) as well as electron-withdrawing substituents (-NO_2_) on the aldehyde, facilitating efficient reactions. The effectiveness of this method is notable, yielding high percentages (92–97%) for the final products.

### 2.3. Characterization of Diethyl 6-Amino-5-Cyano-4-Phenyl-1,4-Dihydropyridine-2,3-Dicarboxylate (**5a**) Using Al(DS)_3_

The structural elucidation of the novel scaffold is accomplished by using various spectroscopic methods such as FTIR, ^1^H NMR, ^13^C NMR, Mass, and elemental analysis. In the series, the IR spectra of compound **5a** reveal three significant absorptions at 3360.7, 2250.6, and 1740.5 cm^−1^ corresponding to the N-H, C≡N, and C=O groups stretching. In ^1^H NMR spectra (500 MHz, CDCl_3_) of compound **5a**, three singulets at *δ* 11.94, 6.59, and 4.54 were observed for NH, NH_2_, and CH, respectively, confirming the formation of 1,4-DHP. The multiplet for 5 protons was at *δ* 7.13–7.27 for the phenyl ring attached at the C-4 position. One quadruplet and one triplet at *δ* 4.17–4.13 and 0.90–0.91 were observed for CH_2_ and CH_3_ protons of ester linkage.

In ^13^C, NMR spectra of molecule **5a**, most down-shielded peaks at *δ* 190.26 and 189.73 ppm were observed for the C=O group. Peaks at *δ* 160.57, 157.91, 154.74, 153.24, and 54.77 ppm were observed for C-2, C-6, C-3, C-5, and C-4 carbon atoms of the main 1,4-DHP skeleton, whereas carbon atoms belonging to the aromatic region were observed at *δ* 136.15 (C-1′), 135.43 (C-2′ and C-6′), 128.34 (C-3′ and C-5′), and 120.71 (C-4′) ppm. The peaks for C≡N and other *sp^3^* carbons were observed at the appropriate position. Furthermore, ESI-MS fragmentation Pattern and Elemental analysis also support the formation of final 1,4-DHP derivatives. The spectral data of compound **5a** strongly corroborates the assigned structure.

### 2.4. Anti-Microbial Activity

The novel synthesized compounds **5a**–**n** underwent testing against two Gram-positive bacteria (*Streptococcus pyogenes* MTCC 442, and *Bacillus subtilis* MTCC 441), three Gram-negative bacteria (*Klebsiella pneumonia* MTCC 3384, *Escherichia coli* MTCC 443, and *Staphylococcus aureus* MTCC 96), as well as three fungal strains (*Aspergillus niger* MTCC 281, *Aspergillus janus* MTCC 2751, and *Aspergillus sclerotiorum* MTCC 1008). Fungal strains were cultivated in malt extract medium prior to inoculation for a duration of 72 h at 28 °C, whereas bacterial samples were grown in nutrient broth for 24 h at 37 °C. Each synthesized chemical underwent triplicate testing after being dissolved in DMSO at concentrations of 2, 4, 8, 16, 32, 64, and 128 g/mL using a serial dilution procedure.

## 3. Experimental Section

### 3.1. Materials and Methods

#### 3.1.1. Chemicals

All chemicals used in this study were obtained from Sigma-Aldrich (St. Louis, MO, USA) which were employed without additional distillation, while the solvents were ordered from Loba Chemie (Mumbai, India).

#### 3.1.2. Analytical Instruments

The digital melting point apparatus was employed to measure the melting point of all the resulting products via the open capillary method. IR spectra of the targeted compound were taken using ATR mode on Perkin Elmer (Waltham, MA, USA) Spectrum II. NMR such as ^1^H and ^13^C are collected on a Bruker (Billerica, MA, USA) Avance NEO 500 MHz NMR spectrometer using DMSO as solvent. Chemical shifts (δ) are accounted for in ppm relative to that of TMS as an internal standard. The mass spectroscopy was recorded on LC-MS Spectrometer Model Q-ToF Micromass Thermo Scientific (Waltham, MA, USA) (FLASH 2000) CHN Elemental Analyser is used for fundamental analysis. The thin layer chromatographic (TLC) technique was used to observe the reaction time as well as to check the purity of the compound, and then the visualization of TLC was performed with the help of a UV chamber. XRD patterns of the dried (lyophilized) samples were captured at room temperature using a Bruker D8 advance. The compounds were exposed to monochromatic Cu-Kα radiation (λ = 1.5418 Å, 50 kV, 40 mA) across the 2θ range between <1 and >150°, with steps of 0.02°. SEM micrographs were obtained utilizing a JSM IT500 scanning electron microscope. Elemental analysis on microscopic sections of the Al(DS)_3_ sample was conducted via EDS. SEM images were acquired under high vacuum mode, ranging from 30 nm (30 kV) to 15.0 nm (1.0 kV).

### 3.2. Synthesis of Aluminum Dodecyl Sulfate

**Scheme 1:**
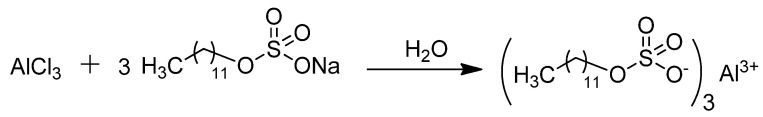
Preparation of Al(DS)_3_ in water.

The preparation of aluminum dodecyl sulfate was conducted following the procedure outlined in the literature (Scheme 1) [28]. Anhydrous AlCl_3_ (0.1 mol) and sodium dodecyl sulfate (SDS) (0.3 mol) were dissolved using the minimum amount of water required. The solutions were gradually diluted with continuous stirring at room temperature, resulting in the precipitation of a colorless solid. This solid was filtered to isolate the solid Al(DS)_3_.

### 3.3. Synthesis of 1,4-DHPs Derivatives (**5a**–**n**)

**Scheme 2:**
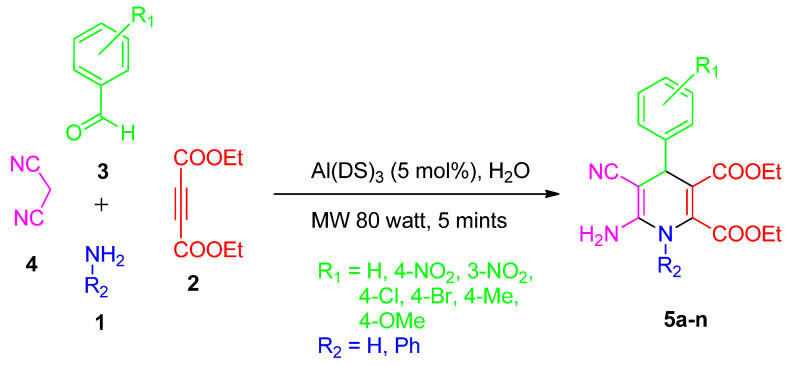
Synthesis of 1,4-DHPs using Al(DS)_3_ in water under microwave radiations.

Using Al(DS)_3_ catalyst (5 mole%), a mixture composed of substituted benzaldehyde (5 mmol), diethyl acetylene dicarboxylate (5 mmol), and either ammonium acetate or aniline (5 mmol), along with malononitrile (5 mmol), was subjected to MW irradiation at 80 watts for 5 min in H_2_O (Scheme 2). The reaction’s progress was monitored using TLC (Merck, Darmstadt, Germany) (EtOAc: Toluene; 8:2). After determining that the reaction had concluded, the mixture was cooled to ambient temperature, filtered, rinsed with water, and subsequently subjected to extraction using ethyl acetate. Subsequently, the resulting solid was recrystallized using ethyl alcohol to yield colorless crystals with an efficiency of 93–97%.

By the optimized reaction conditions, a diversity of 1,4-DHP was prepared using various substituted aldehydes in aqueous micellar solution under MW irradiations at 80 watts for 5 min (Table 3).

*diethyl 6-amino-5-cyano-4-phenyl-1,4-dihydropyridine-2,3-dicarboxylate* (**5a**): Yield 97%, colorless crystals, mp 227–228 °C. IR spectrum, ν, cm^−1^: 3360.7 (N-H **stretch**, -NH_2_), 2250.6 (C≡N), 1740.5 (C=O stretch). ^1^H NMR spectrum, δ, ppm (*J*, Hz): 11.94 (*s*, 1H, NH), 7.13–7.27 (m, 5H, Ar-H), 4.54 (*s*, 1H, CH), 6.59 (*s*, 1H, NH_2_), 4.17–4.13 (*q*, 4H, CH_2_), 0.90–0.91 (*t*, 6H, CH_3_). ^13^C NMR spectrum, δ, ppm: 190.26, 189.73, 160.57, 157.91, 154.74, 153.24, 136.47, 135.43, 128.34, 120.71, 113.47, 54.77, 57.95, 57.96, 13.72, 13.51. Mass spectrum, *m*/*z* (*I*_rel_, %): 342.138 (M + 1), Found: 342.136. Anal. calcd. for C_18_H_19_N_3_O_4_: C, 63.33; H, 5.61; N, 12.31%.

*diethyl 6-amino-5-cyano-4-(4-nitrophenyl)-1,4-dihydropyridine-2,3-dicarboxylate* (**5b**): Yield 96%, colorless crystals, mp 240 °C. IR spectrum, ν, cm^−1^: 3361.1 (N-H stretch, -NH_2_), 2251.3 (C≡N), 1740.7 (C=O stretch). ^1^H NMR spectrum, δ, ppm (*J*, Hz): 12.23 (*s*, 1H, NH), 7.66–7.97 (m, 4H, Ar-H), 4.46 (*s*, 1H, CH), 6.89 (*s*, 1H, NH_2_), 4.16–4.12 (*q*, 4H, CH_2_), 1.19–1.20 (*t*, 6H, CH_3_). ^13^C NMR spectrum, δ, ppm: 190.52, 190.07, 160.85, 158.23, 155.07, 153.57, 136.77, 135.78, 128.63, 121.03, 113.75, 55.03, 58.28, 58.27, 14.03, 13.86. Mass spectrum, *m*/*z* (*I*_rel_, %): 387.123 (M + 1), Found: 387.122. Anal. calcd. for C_18_H_18_N_4_O_6_: C, 55.96; H, 4.70; N, 14.50%.

*diethyl 6-amino-5-cyano-4-(3-nitrophenyl)-1,4-dihydropyridine-2,3-dicarboxylate* (**5c**): Yield 94%, colorless crystals, mp 242 °C. IR spectrum, ν, cm^−1^: 3361.3 (N-H stretch, -NH_2_), 2251.6 (C≡N), 1741.1 (C=O stretch). ^1^H NMR spectrum, δ, ppm (*J*, Hz): 12.10 (*s*, 1H, NH), 7.58–8.12 (m, 4H, Ar-H), 4.81 (*s*, 1H, CH), 6.85 (*s*, 1H, NH_2_), 4.49–4.53 (*q*, 4H, CH_2_), 1.14–1.15 (*t*, 6H, CH_3_). ^13^C NMR spectrum, δ, ppm: 190.41, 189.99, 160.74, 158.17, 154.93, 153.42, 136.64, 135.63, 128.55, 120.97, 113.61, 58.11, 58.10, 54.92, 13.94, 13.74. Mass spectrum, *m*/*z* (*I*_rel_, %): 387.123 (M + 1), Found: 387.122. Anal. calcd. for C_18_H_18_N_4_O_6_: C, 55.96; H, 4.70; N, 14.50%.

*diethyl 6-amino-4-(4-chlorophenyl)-5-cyano-1,4-dihydropyridine-2,3-dicarboxylate* (**5d**): Yield 94%, colorless crystals, mp 237–239 °C. IR spectrum, ν, cm^−1^:3361.6 (N-H stretch, -NH_2_), 2251.7 (C≡N), 1741.5 (C=O stretch). ^1^H NMR spectrum, δ, ppm (*J*, Hz): 12.31 (*s*, 1H, NH), 7.60–7.87 (m, 4H, Ar-H), 4.76 (*s*, 1H, CH), 6.93 (*s*, 1H, NH_2_), 4.09–4.13 (*q*, 4H, CH_2_), 1.16–1.17 (*t*, 6H, CH_3_). ^13^C NMR spectrum, δ, ppm: 190.68, 190.18, 160.96, 158.35, 155.09, 153.65, 136.91, 135.95, 128.81, 121.18, 113.97, 58.35, 58.37, 55.14, 14.26, 14.04. Mass spectrum, *m*/*z* (*I*_rel_, %): 376.099 (M + 1);377.099 (M + 2), Found: 376.098(M + 1); 377.098 (M + 2). Anal. calcd. for C_18_H_18_N_3_BrO_4_: C, 57.53; H, 4.83; N, 11.18%.

*diethyl 6-amino-4-(4-bromophenyl)-5-cyano-1,4-dihydropyridine-2,3-dicarboxylate* (**5e**): Yield 95%, colorless crystals, mp 267–268 °C. IR spectrum, ν, cm^−1^: 3361.9 (N-H stretch, -NH_2_), 2251.9 (C≡N), 1741.6 (C=O stretch). ^1^H NMR spectrum, δ, ppm (*J*, Hz): 12.27 (*s*, 1H, NH), 7.52–7.73 (m, 4H, Ar-H), 4.85 (*s*, 1H, CH), 6.90 (*s*, 1H, NH_2_), 4.51–4.54 (*q*, 4H, CH_2_), 1.13–1.12 (*t*, 6H, CH_3_). ^13^C NMR spectrum, δ, ppm: 190.62, 190.05, 160.91, 158.30, 155.01, 153.61, 136.73, 135.72, 128.58, 121.11, 113.79, 58.31, 58.32, 55.11, 14.20, 13.96. Mass spectrum, *m*/*z* (*I*_rel_, %): 420.048 (M + 1); 421.048 (M + 2), Found: 420.047; 421.047 (M + 2). Anal. calcd. for C_18_H_18_N_3_ClO_4_: C, 51.44; H, 4.32; N, 10.00%.

*diethyl 6-amino-5-cyano-4-p-tolyl-1,4-dihydropyridine-2,3-dicarboxylate* (**5f**): Yield 93%, colorless crystals, mp 230–231 °C. IR spectrum, ν, cm^−1^: 3361.0 (N-H stretch, -NH_2_), 2250.9 (C≡N), 1740.9 (C=O stretch). ^1^H NMR spectrum, δ, ppm (*J*, Hz): 12.15 (*s*, 1H, NH), 7.09–7.41 (m, 4H, Ar-H), 4.87 (*s*, 1H, CH), 6.87 (*s*, 1H, NH_2_), 4.50–4.52 (*q*, 4H, CH_2_), 2.15 (*s*, 3H, CH_3_), 0.89–0.91 (*t*, 6H, CH_3_). ^13^C NMR spectrum, δ, ppm: 190.33, 189.78, 160.79, 158.09, 154.87, 153.48, 136.51, 135.57, 128.42, 120.83, 113.56, 58.01, 58.03, 54.96, 13.95, 13.71. Mass spectrum, *m*/*z* (*I*_rel_, %): 356.153 (M + 1), Found: 356.151. Anal. calcd. for C_19_H_21_N_3_O_4_: C, 64.21; H, 5.96; N, 11.82%.

*diethyl 6-amino-5-cyano-4-(4-methoxyphenyl)-1,4-dihydropyridine-2,3-dicarboxylate* (**5g**): Yield 94%, colorless crystals, mp 238–240 °C. IR spectrum, ν, cm^−1^: 3360.9 (N-H stretch, -NH_2_), 2250.8 (C≡N), 1741.3 (C=O stretch). ^1^H NMR spectrum, δ, ppm (*J*, Hz): 12.01 (*s*, 1H, NH), 7.19–7.49 (m, 4H, Ar-H), 4.79 (*s*, 1H, CH), 6.67 (*s*, 1H, NH_2_), 4.07–4.09 (*q*, 4H, CH_2_), 3.89 (*s*, 3H, OCH_3_), 1.11–1.13 (*t*, 6H, CH_3_). ^13^C NMR spectrum, δ, ppm: 190.09, 189.63, 160.47, 157.83, 154.65, 153.13, 136.38, 135.32, 128.28, 120.64, 113.39, 57.83, 57.81, 54.65, 13.66, 13.50. Mass spectrum, *m*/*z* (*I*_rel_, %): 372.148 (M + 1) Found: 372.146. Anal. calcd. for C_19_H_21_N_3_O_5_: C, 61.45; H, 5.70; N, 11.31%.

*diethyl 6-amino-5-cyano-1,4-diphenyl-1,4-dihydropyridine-2,3-dicarboxylate* (**5h**): Yield 96%, colorless crystals, mp 170–172 °C. IR spectrum, ν, cm^−1^: 3362.3 (N-H stretch, -NH_2_), 2252.5 (C≡N), 1742.1 (C=O stretch). ^1^H NMR spectrum, δ, ppm (*J*, Hz):6.85–8.12 (m, 10H, Ar-H), 4.58 (*s*, 1H, CH), 6.95 (*s*, 1H, NH_2_), 4.40–4.47 (*q*, 4H, CH_2_),2.13–2.14 (*t*, 6H, CH_3_). ^13^C NMR spectrum, δ, ppm: 190.49, 189.99, 160.93, 158.33, 155.12, 153.65, 137.20, 136.87, 136.24, 135.83, 129.15, 128.74, 121.23,121.13, 113.81, 58.36, 58.35, 54.17, 14.12, 13.92. Mass spectrum, *m*/*z* (*I*_rel_, %): 417.169 (M + 1) Found: 417.167. Anal. calcd. for C_24_H_23_N_3_O_4_: C, 69.05; H, 5.55; N, 10.07%.

*diethyl 6-amino-5-cyano-4-(4-nitrophenyl)-1-phenyl-1,4-dihydropyridine-2,3-dicarboxylate* (**5i**): Yield 94%, colorless crystals, mp 171–173 °C. IR spectrum, ν, cm^−1^: 3362.6 (N-H stretch, -NH_2_), 2252.8 (C≡N), 1742.5 (C=O stretch). ^1^H NMR spectrum, δ, ppm (*J*, Hz): 7.33–8.63 (m, 9H, Ar-H), 4.51 (*s*, 1H, CH), 7.31 (*s*, 1H, NH_2_), 4.46–4.50 (*q*, 4H, CH_2_), 2.15–2.16 (*t*, 6H, CH_3_). ^13^C NMR spectrum, δ, ppm: 190.96, 190.47, 161.24, 158.61, 155.48, 153.94, 137.47, 137.17, 136.51, 136.17, 129.31, 129.03, 121.55,121.45, 114.12, 58.68, 58.67, 55.43, 14.43, 14.27. Mass spectrum, *m*/*z* (*I*_rel_, %): 462.154 (M + 1) Found: 462.153. Anal. calcd. for C_24_H_22_N_4_O_6_: C, 62.33; H, 4.79; N, 12.12%.

*diethyl 6-amino-5-cyano-4-(3-nitrophenyl)-1-phenyl-1,4-dihydropyridine-2,3-dicarboxylate* (**5j**): Yield 95%, colorless crystals, mp 270–272 °C. IR spectrum, ν, cm^−1^: 3362.8 (N-H stretch, -NH_2_), 2252.9 (C≡N), 1742.7 (C=O stretch). ^1^H NMR spectrum, δ, ppm (*J*, Hz): 7.27–8.15 (m, 9H, Ar-H), 4.92 (*s*, 1H, CH), 7.27 (*s*, 1H, NH_2_), 4.47–4.51 (*q*, 4H, CH_2_), 1.52–1.53 (*t*, 6H, CH_3_). ^13^C NMR spectrum, δ, ppm: 190.81, 190.41, 161.17, 158.54, 155.36, 153.87, 137.45, 137.04, 136.57, 136.12, 129.26, 128.96, 121.49, 121.40, 114.07, 58.52, 58.51, 55.32, 14.31, 14.19. Mass spectrum, *m*/*z* (*I*_rel_, %): 463.154 (M + 1) Found: 463.153. Anal. calcd. for C_24_H_22_N_4_O_6_: C, 62.33; H, 4.79; N, 12.12%.

*diethyl 6-amino-4-(4-chlorophenyl)-5-cyano-1-phenyl-1,4-dihydropyridine-2,3-dicarboxylate* (**5k**): Yield 96%, colorless crystals, mp 186–187 °C. IR spectrum, ν, cm^−1^: 3363.2 (N-H stretch, -NH_2_), 2253.1 (C≡N), 1742.9 (C=O stretch). ^1^H NMR spectrum, δ, ppm (*J*, Hz): 7.30–7.81 (m, 9H, Ar-H), 4.86 (*s*, 1H, CH), 7.34 (*s*, 1H, NH_2_), 4.48–4.52 (*q*, 4H, CH_2_), 1.78–1.79 (*t*, 6H, CH_3_). ^13^C NMR spectrum, δ, ppm: 191.07, 190.58, 161.33, 158.79, 155.49, 154.16, 137.74, 137.32, 136.89, 136.36, 129.38, 129.27, 121.71, 121.62, 114.35, 58.75, 58.74, 55.54,14.66, 14.41. Mass spectrum, *m*/*z* (*I*_rel_, %): 452.130 (M + 1); 453.130 (M + 1) Found: 452.128 (M + 1); 453.128 (M + 2). Anal. calcd. for C_24_H_22_ClN_3_O_4_: C, 63.79; H, 4.91; N, 9.30%.

*diethyl 6-amino-4-(4-bromophenyl)-5-cyano-1-phenyl-1,4-dihydropyridine-2,3-dicarboxylate* (**5l**): Yield 96%, colorless crystals, mp 151–153 °C. IR spectrum, ν, cm^−1^:3363.5 (N-H stretch, -NH_2_), 2253.3 (C≡N), 1743.1 (C=O stretch). ^1^H NMR spectrum, δ, ppm (*J*, Hz): 7.21–7.69 (m, 9H, Ar-H), 4.95 (*s*, 1H, CH), 7.32 (*s*, 1H, NH_2_), 4.48–4.51 (*q*, 4H, CH_2_), 1.67–1.68 (*t*, 6H, CH_3_). ^13^C NMR spectrum, δ, ppm: 191.02, 190.45, 161.21, 158.72, 155.41, 154.02, 137.52, 137.15, 136.50, 136.19, 129.24, 128.98, 121.68, 121.56, 114.29, 58.72, 58.71, 55.51, 14.60, 14.31. Mass spectrum, *m*/*z* (*I*_rel_, %): 496.079 (M + 1); 497.079 (M + 2) Found: 496.075(M + 1); 497.075 (M + 2). Anal. calcd. for C_24_H_22_BrN_3_O_4_: C, 58.07; H, 4.47; N, 8.47%.

*diethyl 6-amino-5-cyano-1-phenyl-4-p-tolyl-1,4-dihydropyridine-2,3-dicarboxylate* (**5m**): Yield 93%, colorless crystals, mp 286–287 °C. IR spectrum, ν, cm^−1^:3362.5 (N-H stretch, -NH_2_), 2252.1 (C≡N), 1742.6 (C=O stretch). ^1^H NMR spectrum, δ, ppm (*J*, Hz): 6.97–7.93 (m, 9H, Ar-H), 4.96 (*s*, 1H, CH), 7.29 (*s*, 1H, NH_2_), 4.47–4.49 (*q*, 4H, CH_2_), 2.10–2.12 (*t*, 6H, CH_3_), 2.17 (*s*, 3H, CH_3_). ^13^C NMR spectrum, δ, ppm: 190.73, 190.13, 161.11, 158.44, 155.28, 153.89, 137.33, 136.93, 136.32, 135.95, 129.17, 128.82, 121.37, 121.21, 113.96, 58.43, 58.41, 55.37, 14.35, 14.10. Mass spectrum, *m*/*z* (*I*_rel_, %): 432.185 (M + 1) Found: 432.176. Anal. calcd. for C_25_H_25_N_3_O_4_: C, 69.59; H, 5.84; N, 9.74%.

*diethyl 6-amino-5-cyano-4-(4-methoxyphenyl)-1-phenyl-1,4-dihydropyridine-2,3-dicarboxylate* (**5n**): Yield 92%, colorless crystals, mp 253–255 °C. IR spectrum, ν, cm^−1^: 3362.7 (N-H stretch, -NH_2_), 2252.4 (C≡N), 1742.3 (C=O stretch). ^1^H NMR spectrum, δ, ppm (*J*, Hz): 7.08–7.95 (m, 9H, Ar-H), 4.89 (*s*, 1H, CH), 7.05 (*s*, 1H, NH_2_), 4.39–4.45 (*q*, 4H, CH_2_),3.95 (*s*, 3H, OCH_3_), 1.93–1.94 (*t*, 6H, CH_3_). ^13^C NMR spectrum, δ, ppm: 190.47, 190.04, 160.93, 158.25, 155.07, 153.43, 137.19, 136.79, 136.18, 135.71, 128.99, 128.68, 121.16, 121.09, 113.79, 58.23, 58.21, 55.05, 14.08, 13.87. Mass spectrum, *m*/*z* (*I*_rel_, %): 448.179 (M + 1) Found: 448.166. Anal. calcd. for C_25_H_25_N_3_O_5_: C, 67.10; H, 5.63; N, 9.39%.

### 3.4. Plausible Mechanism

**Scheme 3:**
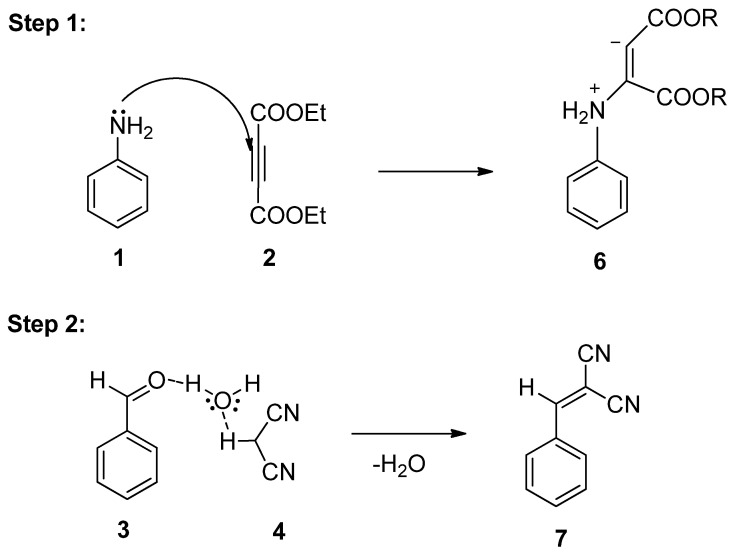

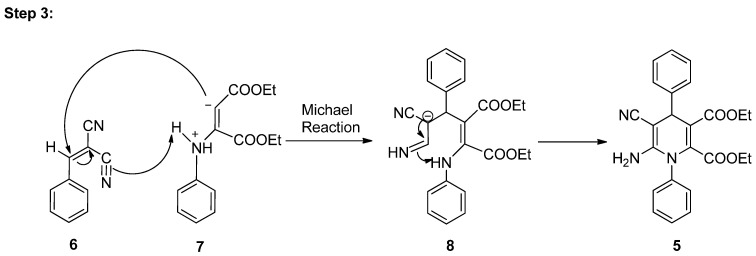
Plausible mechanism for synthesis of 1,4-DHPs using Al(DS)_3_ in water under microwave radiations.

As per literature [32], a conceivable mechanism can be reasonably suggested for the production of pyran pyrazole **5a** from the four-component reaction between aniline **1**, diethyl acetylene dicarboxylate **2**, substituted benzaldehyde **3**, and malononitrile **4** (Scheme 3). The hypothesis suggests that the substituted benzaldehyde **3** may undergo activation by water, leading to increased electrophilicity of the carbonyl carbon. It is suggested that this process involves the establishment of hydrogen bonds between the oxygen atom of the carbonyl group and water molecules, while simultaneously, hydrogen bonds form between the acidic hydrogen of malononitrile and the oxygen of water. Following this, Knoevenagel condensation takes place [33], resulting in the generation of an intermediate **7**. Subsequently, aniline **1** reacts with diethyl acetylene dicarboxylate **2**, resulting in the generation of enolate intermediate **6**. Afterward, the Michael reaction takes place between intermediate **6** and intermediate **7**, which leads to the generation of transient intermediate [34] **8** which undergoes intramolecular cyclization, followed by tautomerization, ultimately resulting in the formation of the target compound, the 1,4-dihydropyridine derivative **9**.

### 3.5. Anti-Microbial Activity

The antimicrobial activities of the synthesized compounds **5a**–**n** were evaluated using the Minimum Inhibitory Concentration (MIC) method. The results were compared to the reference drugs Fluconazole and Amoxicillin, with concentrations of 4 g/mL and 2 g/mL, respectively, in their respective areas of application. Table 4 shows that compound **5a**–**n** exhibits moderate to excellent resistance against the tested strains. 1,4-DHPs with polar electron-withdrawing groups (**5b**, **5c**, **5i**, and **5j**) attached to the phenyl ring at position 4 exhibited effectiveness against all the tested strains, comparable to the standard drugs Amoxicillin (MIC 4 μg/mL) and Fluconazole (MIC 2 μg/mL) this may be due the formation of H-bonds with the different parts of the protein of microbes. However, the resistance efficiency against the tested microbes decreases if the same phenyl ring is substituted with less polar groups such as Me (**5f** and **5m**), OMe (**5g** and **5n**), and Halogens (**5d**, **5e**, **5k**, and **5l**) this is due to their less or no ability of formation of H-Bonds.

## 4. Conclusions

An environmentally green procedure was performed for the synthesis of a novel 1,4-DHP scaffold via a one-pot, four-component reaction using aqueous micellar solution under microwave irradiation by treatment of a mixture of substituted benzaldehyde, diethyl acetylene dicarboxylate, and either ammonium acetate or aniline, along with malononitrile in an equivalent ratio. In summary, the outlined procedure illustrates remarkable efficacy in generating 1,4-DHP derivatives from easily accessible starting materials in a single step, utilizing a micellar solution of Al(DS)_3_ in water. The resulting crops are swiftly attained with adaptability and variety, achieving outstanding yields and purity. This approach demonstrates efficiency in terms of labor, cost-effectiveness, and minimal waste generation while operating under mild reaction conditions. Furthermore, all synthesized 1,4-DHPs display potent activity against the evaluated microbial strains. On a note, it finds that the presence of polar groups such as NO_2_ on the phenyl ring imparts comparable resistance to standard drugs such as Amoxicillin and Fluconazole.

## Figures and Tables

**Table 1:** Impact of solvents on production of the **5a**–**h**.

Solvent	Time (min)	Yield ^a^ (%)
Ethanol	10	87%
Glycerol	8	91%
*p*-TSA + Ethanol	8	89%
PEG	9	90%
Water	15	42%
Al(DS)_3_ + Water	5	97%
SDS + Water	6	92%
CH_3_CN	6	95%
Ethylene glycol	8	94%

^a^ Reactions supported using various solvents. Reactions occurred at 100 °C in MW irradiation.

**Table 2:** Impact of pressure on the percentage yield of **5a**–**h** using Al(DS)_3_ in water.

Watt	Time (min)	Yield ^a^ (%)
65	8	93%
70	7	93%
75	6	95%
80	5	97%
85	4	91%
90	3	88%
95	2	85%

^a^ Yield refers to the combined yield of all crops.

**Table 3:** 1,4-DHP derivatives **5a**–**n** synthesis using Al(DS)_3_ + water for 5 mints under MW radiations.

Compound	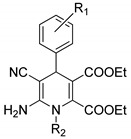		Rf	Yield (%)	Melting Point (°C)	Literature Melting Point (°C)
	R_1_	R_2_				
**5a**	H	H	0.65	97	227–228	-
**5b**	4-NO_2_	H	0.77	96	240	-
**5c**	3-NO_2_	H	0.73	94	242	-
**5d**	4-Cl	H	0.70	94	237–239	-
**5e**	4-Br	H	0.57	95	267–268	-
**5f**	4-Me	H	0.61	93	230–231	-
**5g**	4-OMe	H	0.63	94	238–240	-
**5h**	H	Ph	0.66	96	173–174	170–172 [29]
**5i**	4-NO_2_	Ph	0.69	94	171–173	172–174 [30]
**5j**	3-NO_2_	Ph	0.71	95	270–272	-
**5k**	4-Cl	Ph	0.67	96	186–187	188–189 [31]
**5l**	4-Br	Ph	0.60	96	151–153	152–154 [32]
**5m**	4-Me	Ph	0.61	93	286–287	-
**5n**	4-OMe	Ph	0.65	92	253–255	-

**Table 4:** Anti-microbial activity of synthesized 1,4-DHP derivatives **5a**–**n**.

Compound	Gram (+ve) Bacteria		Gram (−ve) Bacteria			Fungi		
	*B. subtilis*	*S. pyogenes*	*E. coli*	*K. pneumonia*	*S. aureus*	*A. janus*	*A. niger*	*A. sclerotiorum*
**5a**	16	8	8	8	16	8	16	8
**5b**	4	4	4	8	4	4	4	8
**5c**	4	8	4	4	8	8	8	32
**5d**	16	8	16	32	32	16	32	32
**5e**	16	32	-	64	64	32	16	16
**5f**	32	8	16	8	16	16	16	8
**5g**	16	16	8	8	16	8	16	–
**5h**	64	16	32	16	32	32	32	16
**5i**	4	4	4	4	4	4	4	4
**5j**	4	8	8	4	4	8	4	4
**5k**	8	16	16	-	–	16	16	32
**5l**	32	64	128	16	32	16	32	16
**5m**	8	16	64	32	64	16	16	32
**5n**	16	8	8	16	8	16	32	16
**Amoxicillin**	4	4	4	4	4	–	–	–
**Fluconazole**	–	–	–	–	–	2	2	2

## Data Availability

Data are contained within the article and Supplementary Materials.

## Supplementary Materials

The following supporting information can be downloaded at: https://www.mdpi.com/article/10.3390/molecules29051115/s1, Scheme S1: Synthesis of 1,4-DHPs using Al(DS)_3_ in water under microwave radiations; Table S1: Synthesis of 1,4-DHP derivatives 5a-n using Al(DS)3 in water for 5 mints under microwave radiations; Table S2: Indexing of XRD using the Debye Scherrer Method; Figure S1: Energy-Dispersive X-ray Spectroscopy (EDS) data; Figure S2: 1H-NMR of diethyl 6-amino-5-cyano-4-phenyl-1,4-dihydropyridine-2,3-dicarboxylate (**5a**); Figure S3: 1H-NMR of diethyl 6-amino-5-cyano-4-(3-nitrophenyl)-1,4-dihydropyridine-2,3-dicarboxylate (**5c**); Figure S4: 1H-NMR of diethyl 6-amino-5-cyano-1,4-diphenyl-1,4-dihydropyridine-2,3-dicarboxylate (**5h**); Figure S5: 1H-NMR of diethyl 6-amino-5-cyano-4-(3-nitrophenyl)-1-phenyl-1,4-dihydropyridine-2,3-dicarboxylate (**5j**).
