# Supplementary material for: Facile One-Pot Synthesis and Anti-Microbial Activity of Novel 1,4-Dihydropyridine Derivatives in Aqueous Micellar Solution under Microwave Irradiation
Source: Molecules. 2024 Mar 1;29(5):1115. doi: 10.3390/molecules29051115 (PMC10934481; doi:10.3390/molecules29051115)
Supplement: Supplementary file 1 [file molecules-29-01115-s001.zip › molecules-2892597-supplementary.pdf]

Electronic Supplementary Information

Facile One-Pot Synthesis and Anti-Microbial Activity of Novel 1,4-Dihydropyridine Derivatives in Aqueous Micellar Solution under Microwave Irradiation

Asmita Goswami [1], Navneet Kaur [1], Manvinder Kaur [1], Kishanpal Singh [2], Harvinder Singh Sohal [1,*], Haesook Han [3] and Pradip K. Bhowmik [3]

[1] Medicinal and Natural Product Laboratory, Department of Chemistry, Chandigarh University, Mohali 140413, Punjab, India; asmitagoswami3@gmail.com (A.G.)
[2] Department of Chemistry, Punjabi University, Patiala 147002, Punjab, India; kishanpal.3112@gmail.com
[3] Department of Chemistry and Biochemistry, University of Nevada Las Vegas, 4505 S. Maryland Parkway, Box 454003, Las Vegas, NV 89154, USA; hanh3@unlv.nevada.edu (H.H.)
* Correspondence: drharvinder.cu@gmail.com

Abstract: The current study describes a novel and eco-conscious method to synthesize 1,4-dihydropyridine derivatives utilizing an aqueous micellar solution containing $Al(DS)_3$, using readily available starting material. The final products were synthesized with excellent yields within remarkably quick reaction durations, promoting remarkable atom economy and minimizing environmental impacts. The present protocol has several advantages over other methodologies in terms of high yield (up to 97%) with excellent purity. Further, the synthesized 1,4-DHPs exhibit favourable to excellent resistance against examined bacterial and fungal species. Intriguingly, polar groups on the phenyl ring (**5b, 5c, 5i** & **5j**) make the 1,4-DHPs equally potent against the microbes as compared to the standard drugs.

1. Materials & Methods

1.1. Chemicals

The entire chemicals used in this study were got from Sigma Aldrich and were employed without further purification, while the solvents were ordered from Loba Chemie.

1.2. Analytical instruments

The digital melting point apparatus was employed to measure the melting point of all the resulting products *via* open capillary method. IR spectra of the targeted compound were taken using ATR mode on Perkin Elmer Spectrum II. NMR such as ^{1}H and ^{13}C are collected on a Bruker Avance NEO 500 MHz NMR spectrometer using DMSO as solvent. Chemical shifts (δ) are accounted for

in ppm relative to that of TMS as internal standard. The mass spectroscopy was recorded on LC-MS Spectrometer Model Q-ToF Micromass Thermo Scientific (FLASH 2000) CHN Elemental Analyser is used for fundamental analysis. Thin layer chromatographic (TLC) technique was used to observe the reaction time as well as to check the purity of the compound, and then the visualization of TLC is done with the help of a UV chamber. XRD patterns of the dried (lyophilized) samples were captured at room temperature using a Bruker D8 advance. The compounds were exposed to monochromatic Cu-Kα radiation (λ = 1.5418 Å, 50 kV, 40 mA) across the 2θ range between <1 and >150˚, with steps of 0.02˚. SEM micrographs were obtained utilizing a JSM IT500 scanning electron microscope. Elemental analysis on microscopic sections of the $Al(DS)_3$ sample was conducted via EDS. SEM images were acquired under high vacuum mode, ranging from 30 nm (30kV) to 15.0 nm (1.0 kV).

2. Synthesis of 1,4-DHPs derivatives (5a-n)

Using $Al(DS)_3$ catalyst (5 mole%), a mixture composed of substituted benzaldehyde (5 mmol), diethyl acetylene dicarboxylate (5 mmol), and either ammonium acetate or aniline (5 mmol), along with malononitrile (5 mmol), was subjected to MW irradiation at 80 watts for 5 minutes in H_2O **(Scheme 2)**. The reaction's progress was monitored using TLC (EtOAc: Toluene; 8: 2). After determining that the reaction had concluded, the mixture was cooled to ambient temperature, filtered, rinsed with water, and subsequently subjected to extraction using ethyl acetate. Subsequently, the resulting solid was recrystallized using ethyl alcohol to yield colorless crystals with an efficiency of 93-97%.

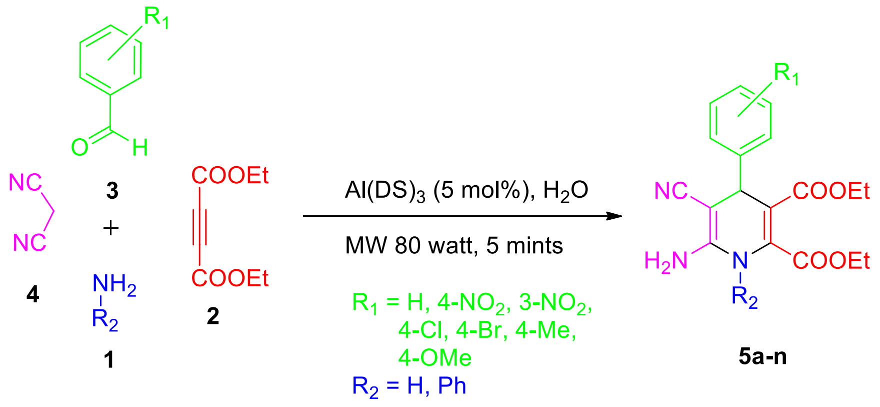

Scheme S1: Synthesis of 1,4-DHPs using $Al(DS)_3$ in water under microwave radiations

Similarly, various other aldehydes **3b-g** will react with diethyl acetylene dicarboxylate, malononitrile, and ammonium acetate or aniline using the same procedure and their formation is monitored with TLC and melting point **(Table 3)**.

Table S1: Synthesis of 1,4-DHP derivatives **5a-n** using $Al(DS)_3$ in water for 5 mints under microwave radiations

**Compound**	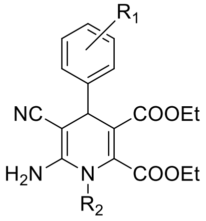		**Rf**	**Yield (%)**	**Melting Point (ºC)**	**Literature Melting Point (ºC)**
	**R_1**	**R_2**				
5a	H	H	0.65	97	227-228	-
5b	4-NO_2	H	0.77	96	240	-
5c	3-NO_2	H	0.73	94	242	-
5d	4-Cl	H	0.70	94	237-239	-
5e	4-Br	H	0.57	95	267-268	-
5f	4-Me	H	0.61	93	230-231	-
5g	4-OMe	H	0.63	94	238-240	-
5h	H	Ph	0.66	96	173-174	170-172 [30]
5i	4-NO_2	Ph	0.69	94	171-173	172-174 [31]
5j	3-NO_2	Ph	0.71	95	270-272	-
5k	4-Cl	Ph	0.67	96	186-187	188-189 [32]
5l	4-Br	Ph	0.60	96	151-153	152-154 [31]
5m	4-Me	Ph	0.61	93	286-287	-
5n	4-OMe	Ph	0.65	92	253-255	-

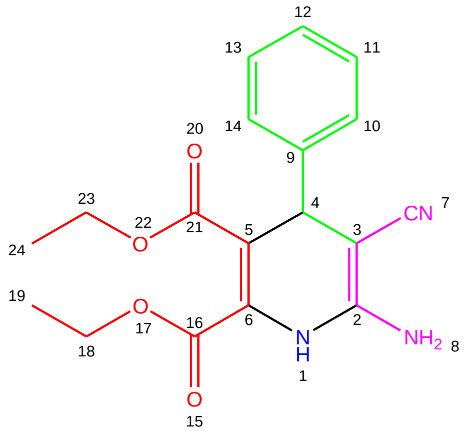

diethyl 6-amino-5-cyano-4-phenyl-1,4-dihydropyridine-2,3-dicarboxylate **(5a):** Yield 97%, colourless crystals, mp 227-228ºC. IR spectrum, υ, cm^{-1}: 3360.7 (N-H stretch, $-NH_2$), 2250.6 (C≡N), 1740.5 (C=O stretch). ^{1}H NMR spectrum, δ, ppm (*J*, Hz): 11.94 (*s*, 1H, NH), 7.13-7.27 (m, 5H, Ar-H), 4.54 (*s*, 1H, CH), 6.59 (*s*, 1H, NH_2), 4.13-4.17 (*q*, 4H, CH_2), 0.90-0.91 (*t*, 6H, CH_3). ^{13}C NMR spectrum, δ, ppm: 190.26, 189.73, 160.57, 157.91, 154.74, 153.24, 136.47, 135.43, 128.34, 120.71, 113.47, 54.77, 57.95, 57.96, 13.72, 13.51. Mass spectrum, *m/z* (I_{rel}, %): 342.138 (M+1), Found: 342.136. Anal. calcd. for $C_{18}H_{19}N_3O_4$: C, 63.33; H, 5.61; N, 12.31%.

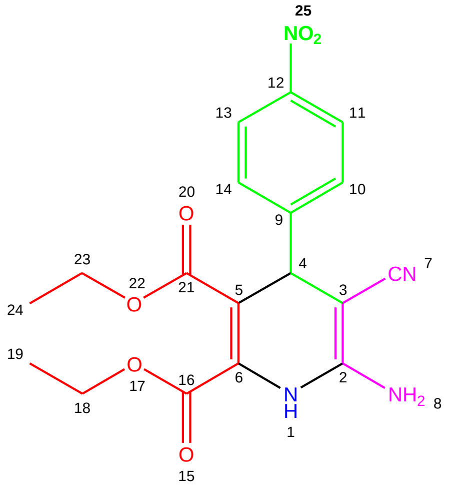

diethyl 6-amino-5-cyano-4-(4-nitrophenyl)-1,4-dihydropyridine-2,3-dicarboxylate **(5b):** Yield 96%, colourless crystals, mp 240ºC. IR spectrum, υ, cm^{-1}: 3361.1 (N-H stretch, $-NH_2$), 2251.3 (C≡N), 1740.7 (C=O stretch). ^{1}H NMR spectrum, δ, ppm (*J*, Hz): 12.23 (*s*, 1H, NH), 7.66-7.97 (m, 4H, Ar-H), 4.46 (*s*, 1H, CH), 6.89 (*s*, 1H, NH_2), 4.16-4.12 (*q*, 4H, CH_2), 1.19-1.20 (*t*, 6H, CH_3). ^{13}C NMR spectrum, δ, ppm: 190.52, 190.07, 160.85, 158.23, 155.07, 153.57, 136.77, 135.78, 128.63, 121.03, 113.75, 55.03, 58.28, 58.27, 14.03, 13.86. Mass spectrum, *m/z* (I_{rel}, %): 387.123 (M+1), Found: 387.122. Anal. calcd. for $C_{18}H_{18}N_4O_6$: C, 55.96; H, 4.70; N, 14.50%.

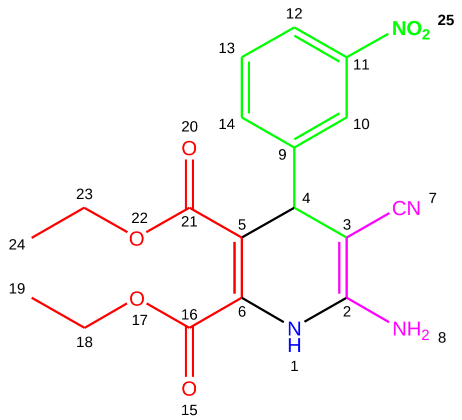

diethyl 6-amino-5-cyano-4-(3-nitrophenyl)-1,4-dihydropyridine-2,3-dicarboxylate **(5c):** Yield 94%, colourless crystals, mp 242ºC. IR spectrum, υ, cm^{-1}: 3361.3 (N-H stretch, $-NH_2$), 2251.6 (C≡N), 1741.1 (C=O stretch).^{1}H NMR spectrum, δ, ppm (*J*, Hz): 12.10 (*s*, 1H, NH), 7.58-8.12 (m, 4H, Ar-H), 4.81 (*s*, 1H, CH), 6.85 (*s*, 1H, NH_2), 4.49-4.53 (*q*, 4H, CH_2), 1.14-1.15 (*t*, 6H, CH_3).^{13}C NMR spectrum, δ, ppm: 190.41, 189.99, 160.74, 158.17, 154.93, 153.42, 136.64, 135.63, 128.55, 120.97, 113.61, 58.11, 58.10, 54.92, 13.94, 13.74. Mass spectrum, *m/z* (I_{rel}, %): 387.123 (M+1), Found: 387.122. Anal. calcd. for $C_{18}H_{18}N_4O_6$: C, 55.96; H, 4.70; N, 14.50%.

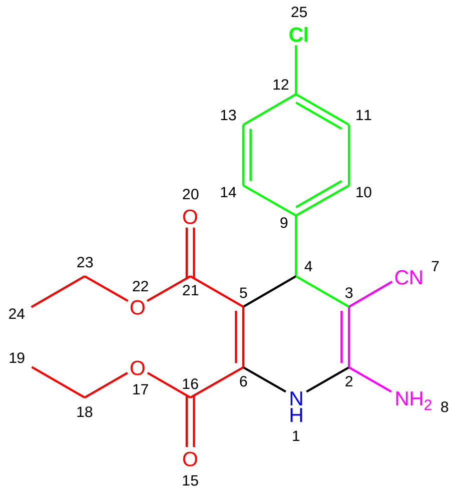

diethyl 6-amino-4-(4-chlorophenyl)-5-cyano-1,4-dihydropyridine-2,3-dicarboxylate **(5d):** Yield 94%, colourless crystals, mp 237-239ºC. IR spectrum, υ, cm^{-1}:3361.6 (N-H stretch, $-NH_2$), 2251.7 (C≡N), 1741.5 (C=O stretch).^{1}H NMR spectrum, δ, ppm (*J*, Hz): 12.31 (*s*, 1H, NH), 7.60-7.87 (m, 4H, Ar-H), 4.76 (*s*, 1H, CH), 6.93 (*s*, 1H, NH_2), 4.09-4.13 (*q*, 4H, CH_2), 1.16-1.17 (*t*, 6H, CH_3).^{13}C NMR spectrum, δ, ppm: 190.68, 190.18, 160.96, 158.35, 155.09, 153.65, 136.91, 135.95, 128.81, 121.18, 113.97, 58.35, 58.37, 55.14, 14.26, 14.04. Mass spectrum, *m/z* (I_{rel}, %): 376.099 (M+1);377.099 (M+2)Found: 376.098(M+1);377.098 (M+2). Anal. calcd. for $C_{18}H_{18}N_3BrO_4$: C, 57.53; H, 4.83; N, 11.18%.

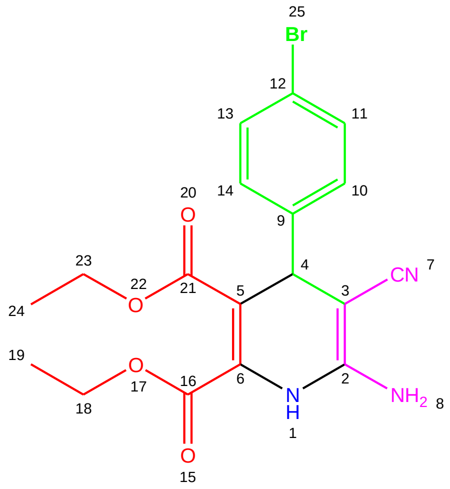

diethyl 6-amino-4-(4-bromophenyl)-5-cyano-1,4-dihydropyridine-2,3-dicarboxylate **(5e):** Yield 95%, colourless crystals, mp 267-268ºC. IR spectrum, υ, cm^{-1}: 3361.9 (N-H stretch, -NH_2), 2251.9 (C≡N), 1741.6 (C=O stretch).^{1}H NMR spectrum, δ, ppm (*J*, Hz): 12.27 (*s*, 1H, NH), 7.52-7.73 (m, 4H, Ar-H), 4.85 (*s*, 1H, CH), 6.90 (*s*, 1H, NH_2), 4.51-4.54 (*q*, 4H, CH_2), 1.13-1.12 (*t*, 6H, CH_3).^{13}C NMR spectrum, δ, ppm: 190.62, 190.05, 160.91, 158.30, 155.01, 153.61, 136.73, 135.72, 128.58, 121.11, 113.79, 58.31, 58.32, 55.11, 14.20, 13.96. Mass spectrum, *m/z* (I_{rel}, %): 420.048 (M+1); 421.048 (M+2), Found: 420.047; 421.047 (M+2). Anal. calcd. for $C_{18}H_{18}N_3ClO_4$: C, 51.44; H, 4.32; N, 10.00%.

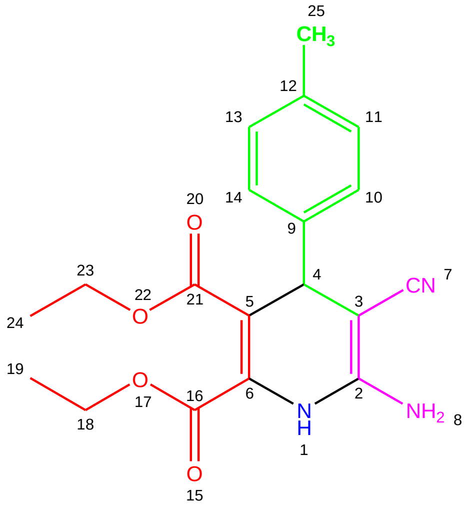

diethyl 6-amino-5-cyano-4-p-tolyl-1,4-dihydropyridine-2,3-dicarboxylate **(5f):** Yield 93%, colourless crystals, mp 230-231ºC. IR spectrum, υ, cm^{-1}: 3361.0 (N-H stretch, -NH_2), 2250.9 (C≡N), 1740.9 (C=O stretch).^{1}H NMR spectrum, δ, ppm (*J*, Hz): 12.15 (*s*, 1H, NH), 7.09-7.41 (m, 4H, Ar-H), 4.87 (*s*, 1H, CH), 6.87 (*s*, 1H, NH_2), 4.50-4.52 (*q*, 4H, CH_2), 2.15 (*s*, 3H, CH_3), 0.89-0.91 (*t*, 6H, CH_3). ^{13}C NMR spectrum, δ, ppm: 190.33, 189.78, 160.79, 158.09, 154.87, 153.48,

136.51, 135.57, 128.42, 120.83, 113.56, 58.01, 58.03, 54.96, 13.95, 13.71. Mass spectrum, *m/z* (I_{rel}, %): 356.153 (M+1), Found: 356.151. Anal. calcd. for $C_{19}H_{21}N_3O_4$: C, 64.21; H, 5.96; N, 11.82%.

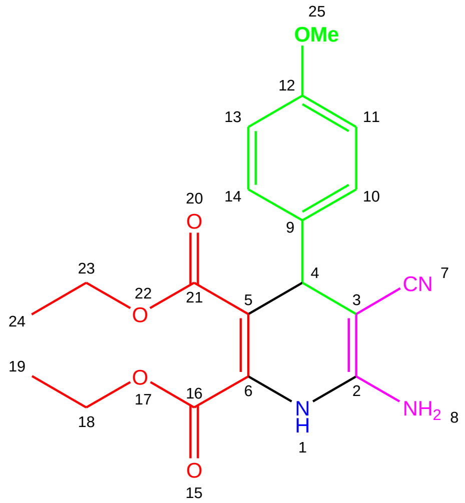

diethyl 6-amino-5-cyano-4-(4-methoxyphenyl)-1,4-dihydropyridine-2,3-dicarboxylate **(5g):** Yield 94%, colourless crystals, mp 238-240ºC. IR spectrum, υ, cm^{-1}: 3360.9 (N-H stretch, $-NH_2$), 2250.8 (C≡N), 1741.3 (C=O stretch).^{1}H NMR spectrum, δ, ppm (*J*, Hz): 12.01 (*s*, 1H, NH), 7.19-7.49 (m, 4H, Ar-H), 4.79 (*s*, 1H, CH), 6.67 (*s*, 1H, NH_2), 4.07-4.09 (*q*, 4H, CH_2), 3.89 (*s*, 3H, OCH_3), 1.11-1.13 (*t*, 6H, CH_3). ^{13}C NMR spectrum, δ, ppm: 190.09, 189.63, 160.47, 157.83, 154.65, 153.13, 136.38, 135.32, 128.28, 120.64, 113.39, 57.83, 57.81, 54.65, 13.66, 13.50. Mass spectrum, *m/z* (I_{rel}, %): 372.148 (M+1) Found: 372.146. Anal. calcd. for $C_{19}H_{21}N_3O_5$: C, 61.45; H, 5.70; N, 11.31%.

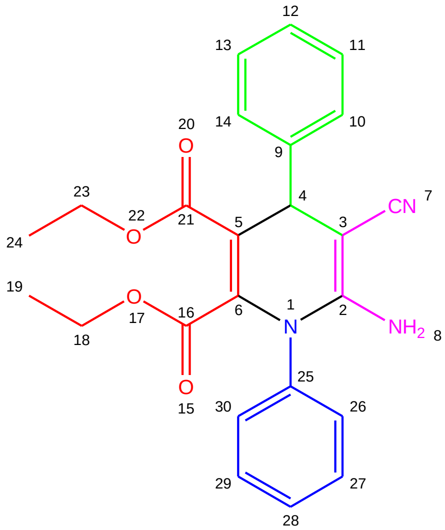

diethyl 6-amino-5-cyano-1,4-diphenyl-1,4-dihydropyridine-2,3-dicarboxylate **(5h):** Yield 96%, colourless crystals, mp 173-174ºC. IR spectrum, υ, cm^{-1}: 3362.3 (N-H stretch, $-NH_2$), 2252.5 (C≡N), 1742.1 (C=O stretch). ^{1}H NMR spectrum, δ, ppm (*J*, Hz):6.85-8.12 (m, 10H, Ar-H), 4.58 (*s*, 1H, CH), 6.95 (*s*, 1H, NH_2), 4.40-4.47 (*q*, 4H, CH_2),2.13-2.14 (*t*, 6H, CH_3). ^{13}C NMR spectrum, δ, ppm: 190.49, 189.99, 160.93, 158.33, 155.12, 153.65, 137.20, 136.87, 136.24, 135.83, 129.15, 128.74, 121.23,121.13, 113.81, 58.36, 58.35, 54.17, 14.12, 13.92. Mass spectrum, *m/z* (I_{rel}, %): 417.169 (M+1) Found: 417.167. Anal. calcd. for $C_{24}H_{23}N_3O_4$: C, 69.05; H, 5.55; N, 10.07%.

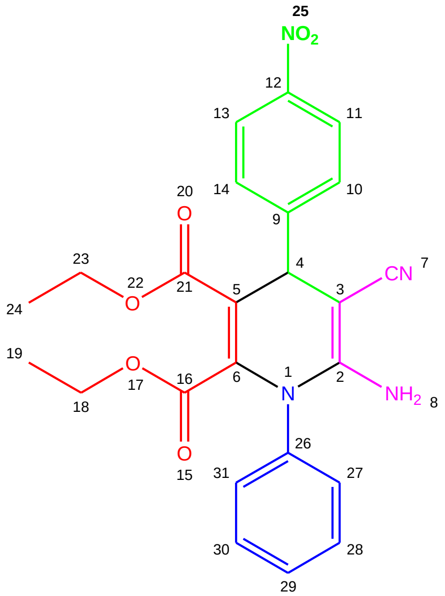

diethyl 6-amino-5-cyano-4-(4-nitrophenyl)-1-phenyl-1,4-dihydropyridine- 2,3-dicarboxylate **(5i):** Yield 94%, colourless crystals, mp 171-173ºC. IR spectrum, υ, cm^{-1}: 3362.6 (N-H stretch, $-NH_2$), 2252.8 (C≡N), 1742.5 (C=O stretch).^{1}H NMR spectrum, δ, ppm (*J*, Hz): 7.33-8.63 (m, 9H, Ar-H), 4.51 (*s*, 1H, CH), 7.31 (*s*, 1H, NH_2), 4.46-4.50 (*q*, 4H, CH_2), 2.15-2.16 (*t*, 6H, CH_3). ^{13}C NMR spectrum, δ, ppm: 190.96, 190.47, 161.24, 158.61, 155.48, 153.94, 137.47, 137.17, 136.51, 136.17, 129.31, 129.03, 121.55,121.45, 114.12, 58.68, 58.67, 55.43, 14.43, 14.27. Mass spectrum, *m/z* (I_{rel}, %): 462.154 (M+1) Found: 462.153. Anal. calcd. for $C_{24}H_{22}N_4O_6$: C, 62.33; H, 4.79; N, 12.12%.

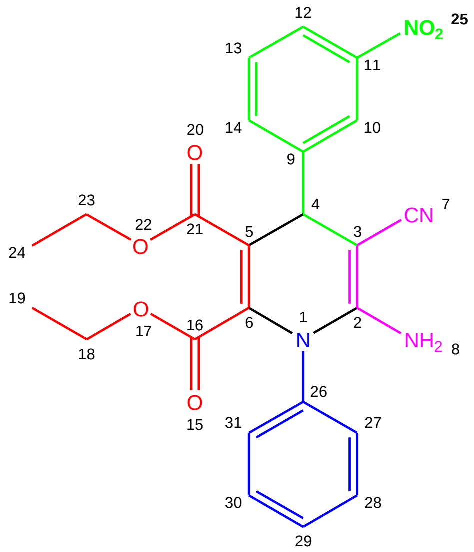

diethyl 6-amino-5-cyano-4-(3-nitrophenyl)-1-phenyl-1,4-dihydropyridine- 2,3-dicarboxylate **(5j):** Yield 95%, colourless crystals, mp 270-272ºC. IR spectrum, υ, cm^{-1}:3362.8 (N-H stretch, $-NH_2$), 2252.9 (C≡N), 1742.7 (C=O stretch).^{1}H NMR spectrum, δ, ppm (*J*, Hz): 7.27-8.15 (m, 9H, Ar-H), 4.92 (*s*, 1H, CH), 7.27 (*s*, 1H, NH_2), 4.47-4.51 (*q*, 4H, CH_2), 1.52-1.53 (*t*, 6H, CH_3). ^{13}C NMR spectrum, δ, ppm: 190.81, 190.41, 161.17, 158.54, 155.36, 153.87, 137.45, 137.04, 136.57, 136.12, 129.26, 128.96, 121.49, 121.40, 114.07, 58.52, 58.51, 55.32, 14.31, 14.19. Mass spectrum, *m/z* (I_{rel}, %): 463.154 (M+1) Found: 463.153. Anal. calcd. for $C_{24}H_{22}N_4O_6$: C, 62.33; H, 4.79; N, 12.12%.

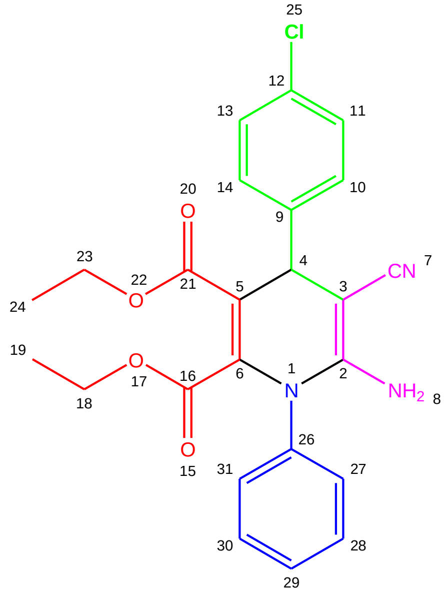

diethyl 6-amino-4-(4-chlorophenyl)-5-cyano-1-phenyl-1,4-dihydropyridine- 2,3-dicarboxylate **(5k):** Yield 96%, colourless crystals, mp 186-187ºC. IR spectrum, υ, cm^{-1}: 3363.2 (N-H stretch, -

NH_2), 2253.1 (C≡N), 1742.9 (C=O stretch).^{1}H NMR spectrum, δ, ppm (*J*, Hz): 7.30-7.81 (m, 9H, Ar-H), 4.86 (*s*, 1H, CH), 7.34 (*s*, 1H, NH_2), 4.48-4.52 (*q*, 4H, CH_2), 1.78-1.79 (*t*, 6H, CH_3). ^{13}C NMR spectrum, δ, ppm: 191.07, 190.58, 161.33, 158.79, 155.49, 154.16, 137.74, 137.32, 136.89, 136.36, 129.38, 129.27, 121.71, 121.62, 114.35, 58.75, 58.74, 55.54,14.66, 14.41. Mass spectrum, *m/z* (I_{rel}, %): 452.130 (M+1);453.130 (M+1) Found: 452.128 (M+1); 453.128 (M+2). Anal. calcd. for $C_{24}H_{22}ClN_3O_4$: C, 63.79; H, 4.91; N, 9.30%.

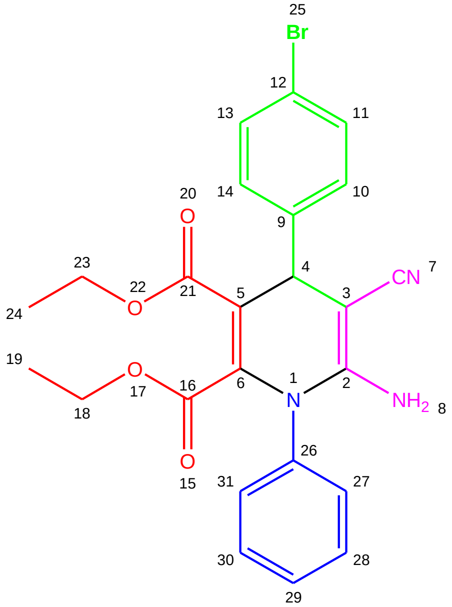

diethyl 6-amino-4-(4-bromophenyl)-5-cyano-1-phenyl-1,4-dihydropyridine- 2,3-dicarboxylate **(5l):** Yield 96%, colourless crystals, mp 151-153ºC. IR spectrum, υ, cm^{-1}:3363.5 (N-H stretch, -NH_2), 2253.3 (C≡N), 1743.1 (C=O stretch).^{1}H NMR spectrum, δ, ppm (*J*, Hz): 7.21-7.69 (m, 9H, Ar-H), 4.95 (*s*, 1H, CH), 7.32 (*s*, 1H, NH_2), 4.48-4.51 (*q*, 4H, CH_2), 1.67-1.68 (*t*, 6H, CH_3). ^{13}C NMR spectrum, δ, ppm: 191.02, 190.45, 161.21, 158.72, 155.41, 154.02, 137.52, 137.15, 136.50, 136.19, 129.24, 128.98, 121.68, 121.56, 114.29, 58.72, 58.71, 55.51, 14.60, 14.31. Mass spectrum, *m/z* (I_{rel}, %): 496.079 (M+1); 497.079 (M+2) Found: 496.075(M+1); 497.075 (M+2). Anal. calcd. for $C_{24}H_{22}BrN_3O_4$: C, 58.07; H, 4.47; N, 8.47%.

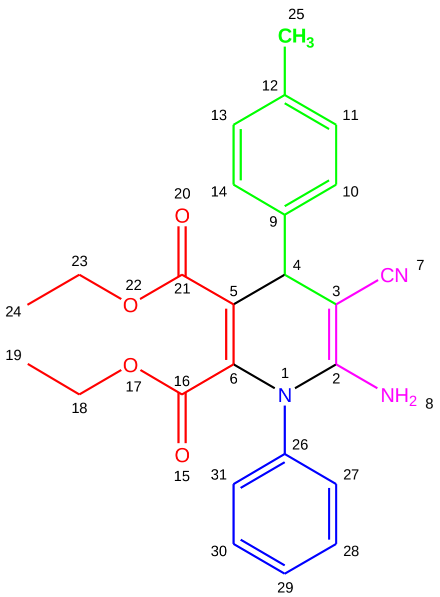

diethyl 6-amino-5-cyano-1-phenyl-4-p-tolyl-1,4-dihydropyridine-2,3-dicarboxylate **(5m):** Yield 93%, colourless crystals, mp 286-287ºC. IR spectrum, υ, cm^{-1}:3362.5 (N-H stretch, -NH_2), 2252.1 (C≡N), 1742.6 (C=O stretch).^{1}H NMR spectrum, δ, ppm (*J*, Hz): 6.97-7.93 (m, 9H, Ar-H), 4.96 (*s*, 1H, CH), 7.29 (*s*, 1H, NH_2), 4.47-4.49 (*q*, 4H, CH_2), 2.10-2.12 (*t*, 6H, CH_3), 2.17 (*s*, 3H, CH_3).^{13}C NMR spectrum, δ, ppm: 190.73, 190.13, 161.11, 158.44, 155.28, 153.89, 137.33, 136.93, 136.32, 135.95, 129.17, 128.82, 121.37, 121.21, 113.96, 58.43, 58.41, 55.37, 14.35, 14.10. Mass spectrum, *m/z* (I_{rel}, %): 432.185 (M+1) Found: 432.176. Anal. calcd. for $C_{25}H_{25}N_3O_4$: C, 69.59; H, 5.84; N, 9.74%.

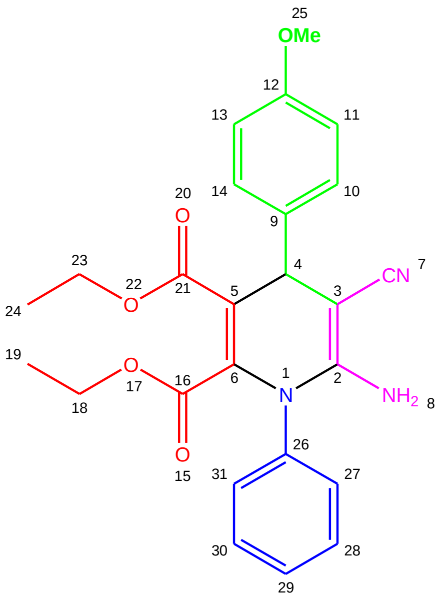

diethyl 6-amino-5-cyano-4-(4-methoxyphenyl)-1-phenyl-1,4-dihydropyridine-2,3-dicarboxylate **(5n):** Yield 92%, colourless crystals, mp 253-255ºC. IR spectrum, υ, cm^{-1}: 3362.7 (N-H stretch, -

NH_2), 2252.4 (C≡N), 1742.3 (C=O stretch).^{1}H NMR spectrum, δ, ppm (*J*, Hz): 7.08-7.95 (m, 9H, Ar-H), 4.89 (*s*, 1H, CH), 7.05 (*s*, 1H, NH_2), 4.39-4.45 (*q*, 4H, CH_2),3.95 (*s*, 3H, OCH_3), 1.93-1.94 (*t*, 6H, CH_3).^{13}C NMR spectrum, δ, ppm: 190.47, 190.04, 160.93, 158.25, 155.07, 153.43, 137.19, 136.79, 136.18, 135.71, 128.99, 128.68, 121.16, 121.09, 113.79, 58.23, 58.21, 55.05, 14.08, 13.87. Mass spectrum, *m/z* (I_{rel}, %): 448.179 (M+1) Found: 448.166. Anal. calcd. for $C_{25}H_{25}N_3O_5$: C, 67.10; H, 5.63; N, 9.39%.

3. Indexing of XRD

Table S2: Indexing of XRD using the Debye Scherrer Method

**S. No.**	**2θ**	**θ (radians)**	**$\sin^2 \theta$**	**$\sin^2 \theta_n / \sin^2 \theta_1$**	**$h^2 + k^2 + l^2$**	**$h^2 + k^2 + l^2$**	**hkl**
**1**	6.596	0.115122	0.114615	1	2	2	110
**2**	7.184	0.125384	0.12473	1.088253	2.176505	2	110
**3**	8.777	0.153188	0.151998	1.326155	2.652309	3	111
**4**	10.965	0.191375	0.189064	1.649556	3.299112	3	111
**5**	13.166	0.22979	0.225809	1.970143	3.940287	4	200
**6**	17.587	0.306951	0.297577	2.596309	5.192619	5	210
**7**	20.387	0.35582	0.341356	2.978276	5.956552	6	211
**8**	20.674	0.360829	0.345762	3.016713	6.033425	6	211
**9**	21.875	0.381791	0.364022	3.176034	6.352069	6	211

4. SEM Characterization

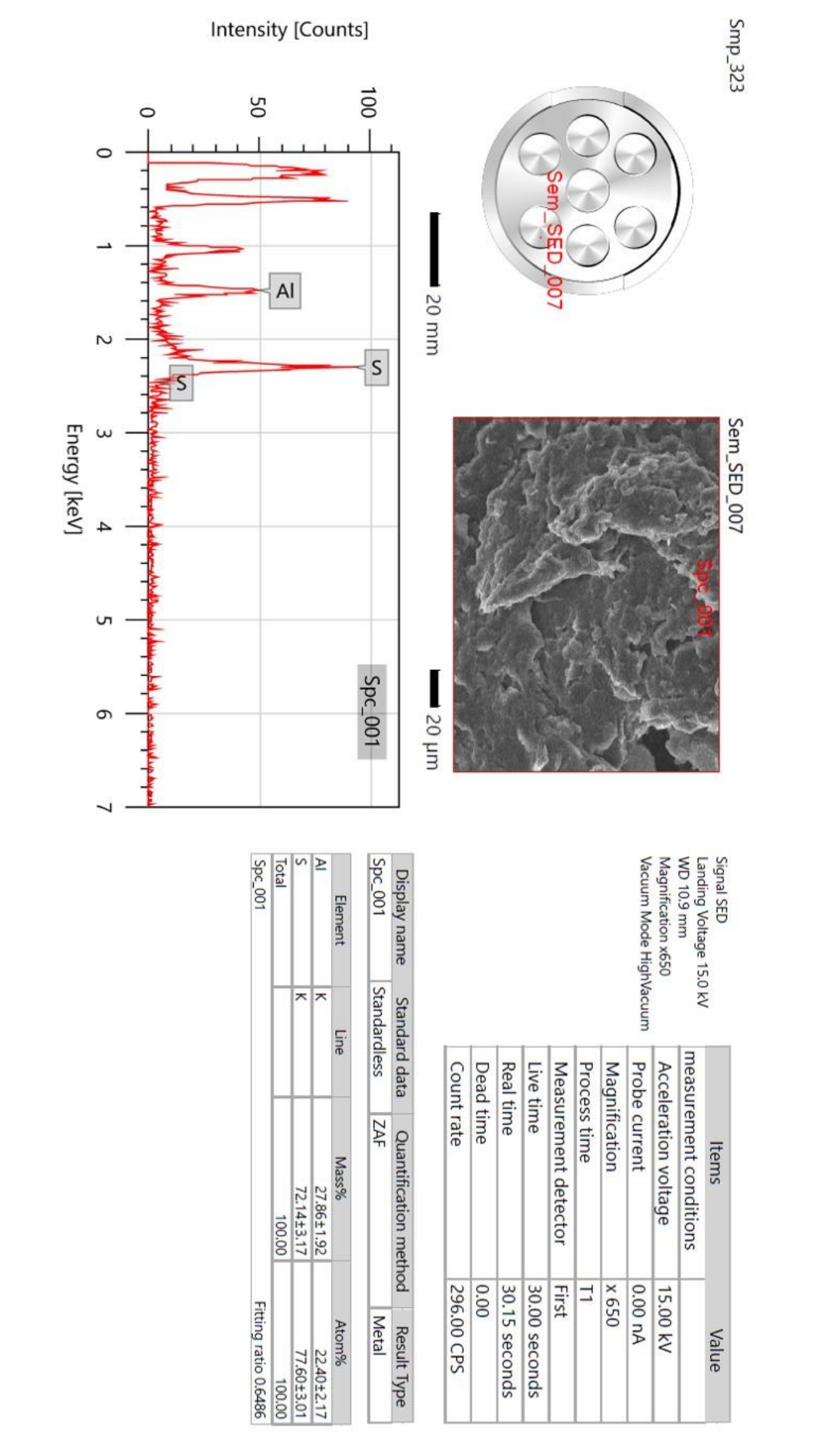

Figure S1: Energy-Dispersive X-ray Spectroscopy (EDS) data

5. ^{1}H-NMR Characterization

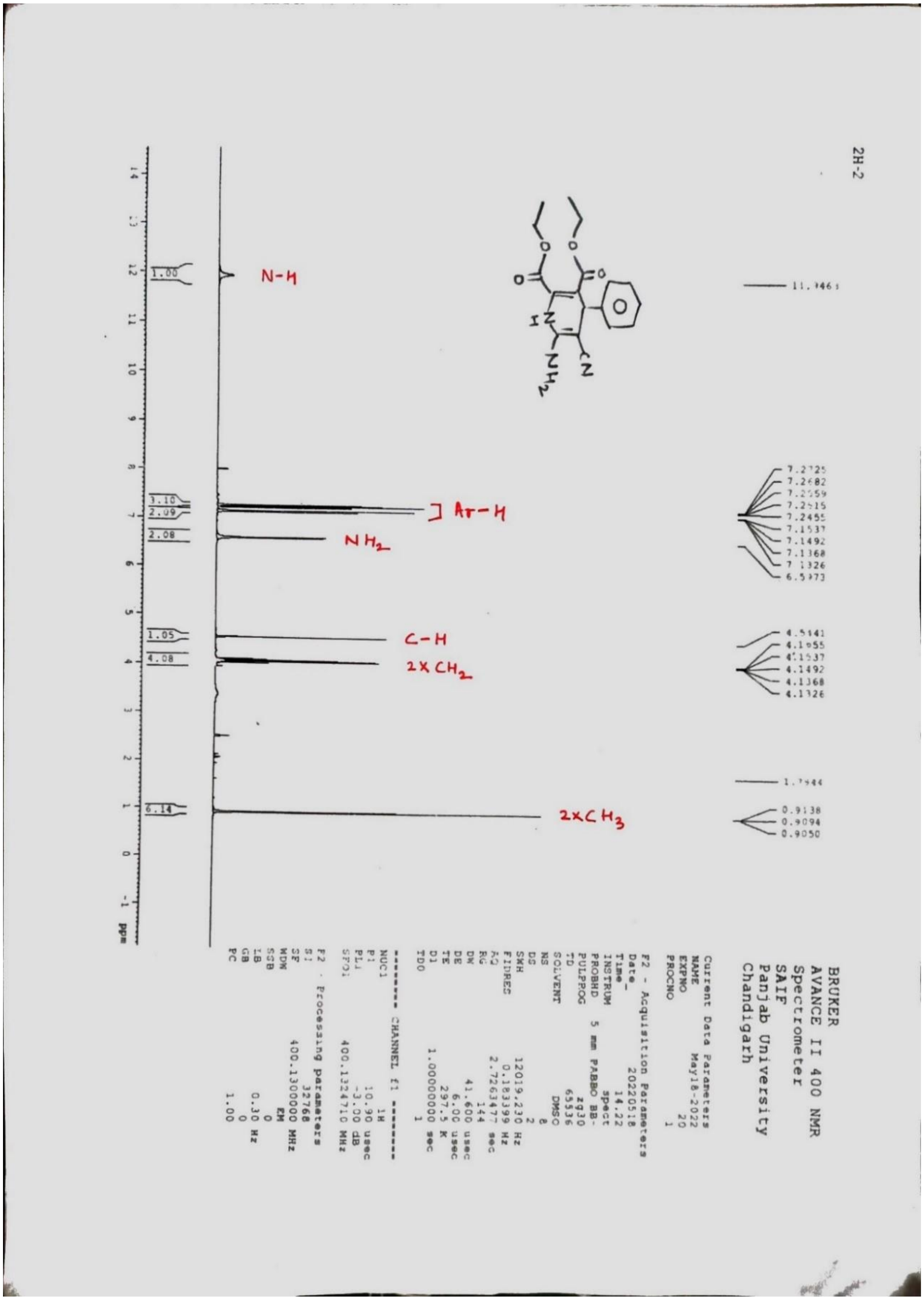

Figure S2: ^{1}H-NMR of *diethyl 6-amino-5-cyano-4-phenyl-1,4-dihydropyridine-2,3-dicarboxylate* **(5a)**

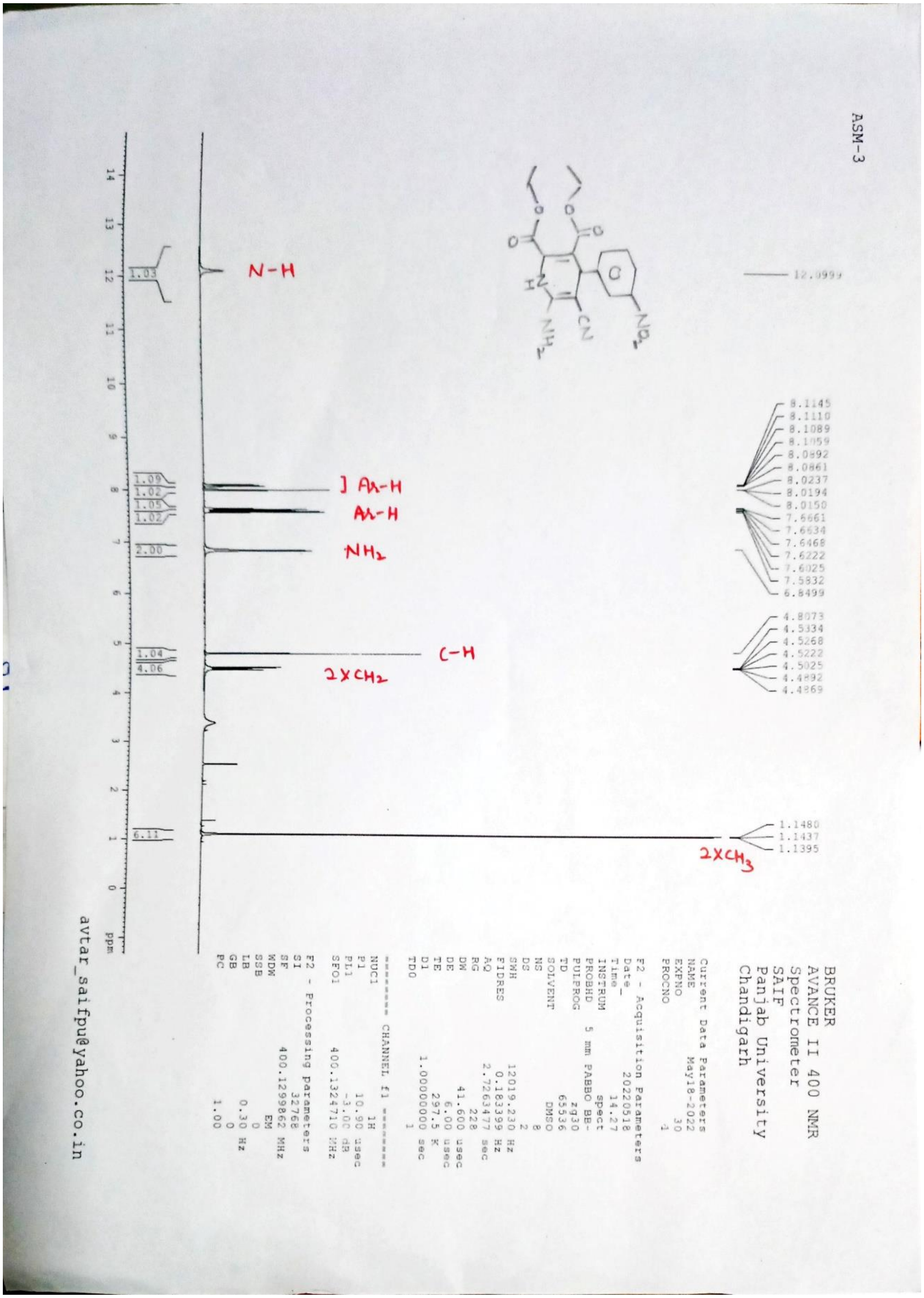

Figure S3: ^{1}H-NMR of *diethyl 6-amino-5-cyano-4-(3-nitrophenyl)-1,4-dihydropyridine-2,3-dicarboxylate* **(5c)**

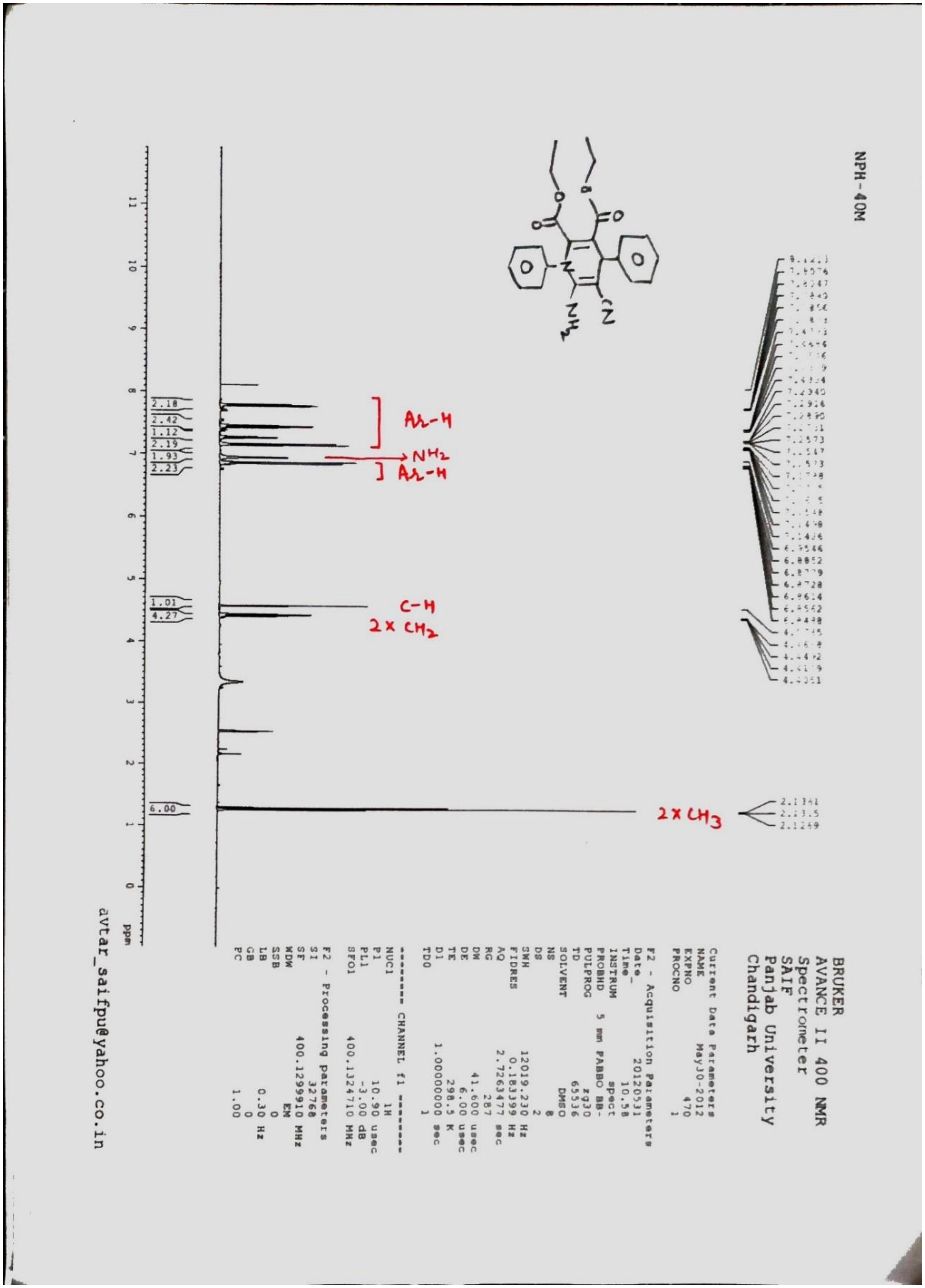

Figure S4: ^{1}H-NMR of *diethyl 6-amino-5-cyano-1,4-diphenyl-1,4-dihydropyridine-2,3-dicarboxylate* **(5h)**

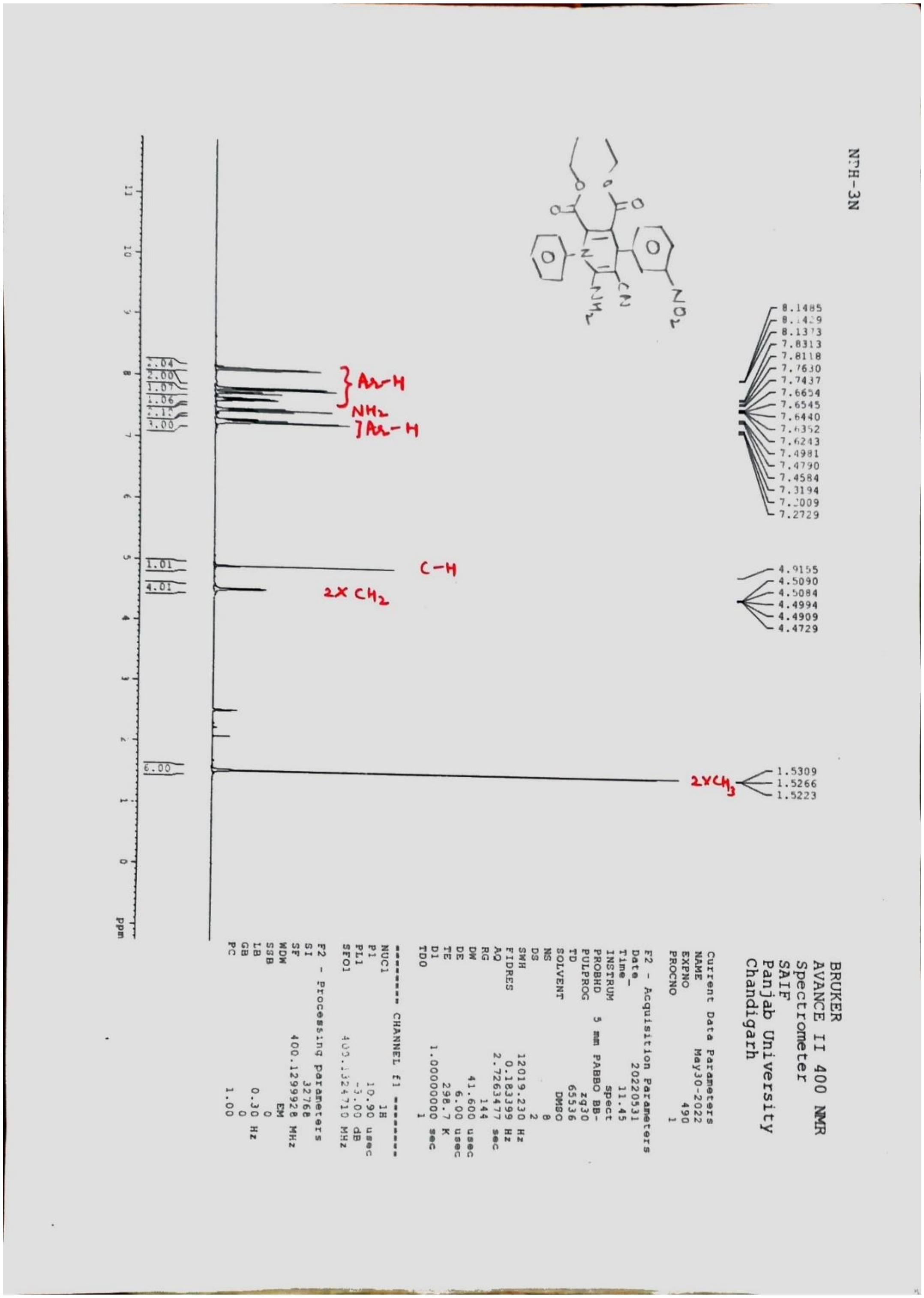

Figure S5: ^{1}H-NMR of *diethyl 6-amino-5-cyano-4-(3-nitrophenyl)-1-phenyl-1,4-dihydropyridine- 2,3-dicarboxylate* **(5j)**
